# A Molecular Dynamics Simulation Based Investigation of the Proton Conductivity of Anhydrous Pyrazole Doped Poly(Vinylphosphonic Acid) Composite System

**DOI:** 10.3390/polym12122906

**Published:** 2020-12-04

**Authors:** Yu-Ren Huang, Chung-Te Chang Chien, Cheng-Lung Chen

**Affiliations:** 1 Department of Applied Science, Naval Academy, Kaohsiung 813, Taiwan; 2 Department of Chemistry, National Sun Yat-Sen University, Kaohsiung 804, Taiwan; allanhuston@gmail.com (C.-T.C.C.); chen1@mail.nsysu.edu.tw (C.-L.C.)

**Keywords:** proton transport, anhydrous proton exchange membrane, hydrogen bond, molecular dynamics simulations, high temperature fuel cell

## Abstract

With the recognition of the multiple advantages of proton transport membranes that can operate under anhydrous conditions and offer promising opportunities as fuel cells working at high temperatures, a number of such membranes have been developed, but the proton transport mechanism of these materials has not been fully understood. In this work, a theoretical investigation based on molecular dynamics simulations is carried out on a system that is very similar to a real anhydrous proton transport membrane. The location and type of hydrogen bonds have been precisely identified by intermolecular pair correlation functions. Furthermore, analysis of the proton coordination numbers shows that more protons are located in the neighborhood of the oxygen atoms of poly(vinyl phosphonate anion) than in the neighborhood of the nitrogen atoms of pyrazole. The proton conductivity, 1.06 × 10^−3^ Scm^−1^, is obtained by the self-diffusion coefficient of the protons at 423 K, which is reasonably close to the experimentally measured value, 2 × 10^−4^ Scm^−1^. In addition, the analysis of the proton trajectories provides us with the proton transfer mechanism in an anhydrous membrane: (a) proton hopping between the oxygen atoms of poly(vinyl phosphonate anion) and (b) proton hopping between two pyrazole molecules. Therefore, the network of the hydrogen bond is the pathway to transport protons via the processes of hydrogen bond forming and breaking.

## 1. Introduction

Polymer electrolyte membrane fuel cells (PEMFCs), or proton exchange membrane fuel cells, are a type of fuel cell being developed mainly for transport vehicles, portable devices and stationary applications. They have emerged as one of the most promising candidates in power conversion devices due to advantages such as efficient power generation, less emission of pollution and a minimized design [1,2]. The proton electrolyte membrane, or proton exchange membrane, is the most critical component in PEMFCs because it plays multiple roles in an operating PEM fuel cell: as a medium for proton conduction, a gas separator between anode and cathode and a stopper for blocking fuel crossover. At present, the Nafion membrane manufactured by Dupont is the best known commercial product based on poly(perfluorosulfonic acid) [3,4,5,6,7,8]. In the Nafion membrane, the mobility of protons is directly affected by the quantity of water molecules inside the membrane. Hence, the conductivity of proton drops significantly with a decrease in humidity when a PEMFC is operating at high temperatures [5,6,7,8]. In addition to being used as a PEM, Nafion has many other valuable applications. For instance, in a recent interesting study [9], Nafion was demonstrated to increase the performance of enzymatic electrochemical sensors where enzyme–PQQ-dependent glucose dehydrogenase immobilized within Nafion membrane exhibits greater catalytic activity and possesses other interesting analytical characteristics. However, besides its high cost, the fact that the proton conductivity of Nafion relies on sufficient hydration results in limitations to the use of Nafion membranes in applications where fuel cells are running at high temperatures.

To date, more and more research focusing on anhydrous or extremely low humidity PEMs based on hydrocarbon polymers has been conducted under high temperature conditions (100–200 °C) [10,11,12,13,14,15,16]. These PEMs possess a number of attractive advantages, such as improving the performance of PEMFCs at high temperatures, increasing the tolerance of electrode to impure fuels and integrating into cogeneration systems [3,17]. To date, a series of acid-doped polymers have been widely studied as the candidate of membranes for high temperature proton exchange membrane fuel cells (HT-PEMFCs), such as poly(vinylpyrrolidone) (PVP) [18,19], poly(ether ether ketone) (SPEEK) [20,21] and polybenzimidazole (PBI) [22,23,24,25]. In particular, the acid-doped PBI system has been extensively investigated due to its high proton conductivity, thermal stability, good mechanical strength and low cost [3]. More importantly, the membranes are still able to maintain normal function at temperatures as high as 200 °C [20,26,27].

In the systems of anhydrous or low humid acid-doped polymers operating at higher temperatures (100–200 °C), it has been confirmed that the transport of proton is mainly based on the Grotthuss mechanism (non-vehicular mechanism) [11,22]. In this mechanism, protons can transfer from one site to the next without relying on any diffusible vehicular molecules, such as water molecules. It is much different from the proton transport in Nafion systems, which largely depend on vehicular mechanism [3,28,29]. In the Grotthuss mechanism, the transport of protons depends on the existence of a donor and acceptor of protons. Therefore, for a PEM to operate with this mechanism, it must have both a donor and acceptor. 

Organic heterocycles, such as imidazole, pyrazole and 1-methylimidazole, are well known to be excellent in assisting proton transport. The primary reasons are that, not only can the nitrogen of the heterocycle play the role as a mediator of protons, but also the heterocycle can act as a proton carrier. Therefore, heterocycle-doped polymers with Bronsted acid (acid–base composite) have been also considered to be a promising candidate material for proton transport membranes [12,30,31]. Yamada et al. investigated a series of acid–base composites by mixing poly(vinylphosphonic acid) with heterocycle molecules, such as imidazole, pyrazole, and 1-methylimidazole. The maximal conductivity of proton for heterocycle-doped poly(vinylphosphonic acid) under 150 °C can approach 7 × 10^−3^ Scm^−1^, 8 × 10^−4^ Scm^−1^ and 1 × 10^−3^ Scm^−1^ for imidazole, pyrazole and 1-methylimidazole in an anhydrous condition, respectively [12]. In addition, Yan et al. employed molecular dynamics (MD) simulations to investigate similar systems as mentioned above and concluded that a hydrogen-bond network provides the pathway to promote the transport of protons [32]. However, their simulation systems based on poly(vinylphosphonic acid) oligomers are much different from the realistic systems based on poly(vinylphosphonic acid) polymers. Furthermore, the structure of deprotonation has been found in base doped poly(vinylphosphonic acid), an indication of the formation of free protons, which is unacceptable [33,34,35,36].

Therefore, in this study, a simulation system corresponding to more realistic situations, including an organic base heterocycle pyrazole (Py), poly(vinyl phosphonate anion) and protons, was set up to investigate the behavior of protons at a higher temperature (150 °C). A theoretical estimated value of proton conductivity, 1.06 × 10^−3^ S cm^−1^, close to the experimental conductivity value, 2 × 10^−4^ Scm^−1^, was obtained. Furthermore, the analysis of the coordination number and the trajectories of protons was carried out to help us understand proton transport in the network of hydrogen bonds under anhydrous conditions. 

## 2. Computation Methods

### 2.1. Force Field

The simulation system consists of H, C, N, O, and P atoms. A generic force field, Dreiding, that we chose is employed in our work because it is most suitable in predicting the structures and dynamics of organic compounds involving the nonmetallic main-group elements. In addition, Dreiding force field also can result in reasonably accurate barriers and geometries in various organic systems [37]. Moreover, it further provides a proper potential model to predict the simulation systems of polymers mixing with small molecules or charged species accurately [38,39]. In the present study, this force field, Dreiding, is available through Materials Studio 6.0 (Accelrys commercial software).

**Figure 1:**
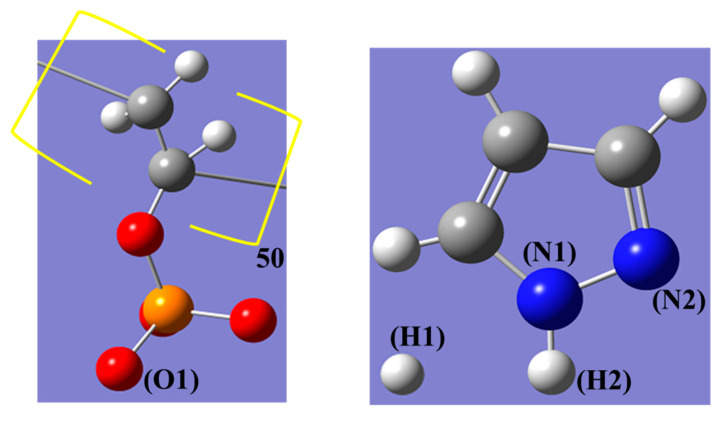
Atomic site symbols of poly(vinyl phosphonate anion) (PVPA), pyrazole (Py) and proton.

The chain of poly(vinyl phosphonate anion) (PVPA) was built from the “Build Polymers_TAB” software in Materials Studio 6.0. The “Amorphous Cell” of Accelrys provides a way to construct the model including four poly(vinyl phosphonate anion) polymer chains each containing 50 repeat units, 400 protons (H^+^) and 1618 pyrazole (Py) molecules. The construction of simulation system was based on the study by Yamada et al. [12] The atomic site symbols of repeat unit of PVPA, Py and H^+^ are shown in Figure 1. We employed quantum chemistry (QC) calculations to evaluate the intermolecular charge transfer in poly(vinyl phosphonate anion) and along pyrazole groups. In QC calculations, density functional theory (DFT) was employed. We adopted Becke’s three-parameter hybrid functional combined with the Lee–Yang–Parr correlation functional method (B3LYP).

In the beginning, the simulation cell was constructed by inserting the molecules randomly.

**Figure 2:**
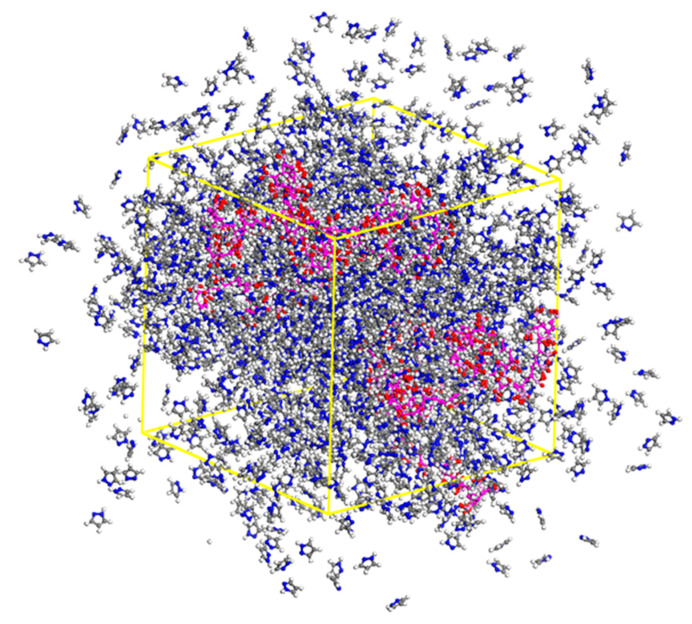
The simulation cell of mixing poly(vinyl phosphonate anion), pyrazole (Py) and proton.

The initial density of simulation cell was set to 0.05 g/cm^3^, and the cell size of box was 76 × 76 × 76 Å^3^. The geometry optimization of the cell was executed with a “smart” algorithm and then molecular dynamics simulation calculations were carried out with periodic boundary conditions until the system reached thermal equilibration. After that, we squeezed the cell slightly and repeated the steps as mentioned until the density of the system reached 1.649 g/cm^3^ and the final size of the simulation cell was 51 × 51 × 51 Å^3^, as shown in Figure 2. After the equilibrium density was reached, a 140 ps NVT ensemble dynamics was run while maintaining volume and temperature at a constant by using the Forcite Module. The time step was 1 fs and the setting of the temperature was 423 K. The cut-off radius of the non-bonded interactions was set to 18.5 Å. Ewald summation was employed for both van der Waals and Coulombic interactions [40,41].

### 2.2. Molecular Dynamics

In the dynamics simulation, the mobile trajectory of proton can generate mean square displacement (MSD), the average square of displacement where a particle moves from time 0 to time *t*. The self-diffusion coefficient of particle was calculated with the slop of MSD as a function of *t* in the form:

(1) $$D=\frac{1}{6N}\underset{t\to \infty }{lim}\frac{d}{dt}\overset{N}{\underset{i=1}{\sum }}{\left|r_{i}\left(t\right)-r_{i}\left(0\right)\right|}^{2}$$

in which *D* represents the diffusion coefficient, *N* represents the number of diffusible particles, and *r_i_* represents the position vector of particles at time event, respectively.

The diffusion coefficient of proton can be employed to generate the proton conductivity (*σ*, in units of Siemens per centimeter) by Equation (2) as below:

(2) $$\sigma =\frac{Nz^{2}e^{2}}{Vk_{B}T}D_{proton}$$

in which *N* represents the number of protons, *Z* the charge of proton (+1 in this study), *e* the elementary charge (1.6 × 10^−19^ C in this study), *D_proton_* the diffusion coefficient of proton, *V* the volume of the simulation cell, *k_B_* the Boltzmann’s constant and *T* the absolute temperature, respectively [42,43]. The interaction of specific two particles was studied by the calculation of intermolecular pair correlation function or radial distribution function (RDF),$\;g_{xy}\left(r\right)$ given below, which represents the probability of detecting particle *y* separated from particle *x* at distance *r* by comparing the local density with the bulk density [42,43].

(3) $$g_{xy}\left(r\right)=\frac{N_{xy}\left(r\right)V}{4\pi r^{2}N_{x}N_{y}dr}$$

The coordination number can be obtained by using $g_{xy}\left(r\right)$, in which *N_xy_* represents the total number of *x* particles coordinated with *y* particles within a distance *r*, *N_x_* the total number of *x* particles, *N_y_* the total of *y* particles, *V* the volume of the simulation cell, and $g_{xy}\left(r\right)\;$ the pair correlation function between two specific particles *x* and *y*, respectively [42].

(4) $$N_{xy}\left(r\right)=4\pi \frac{N_{y}}{V}\overset{r}{\underset{0}{\int }}g_{xy}\left(r\right)r^{2}dr\;$$

## 3. Results and Discussion

### 3.1. Dynamics Properties of Protons

**Figure 3:**
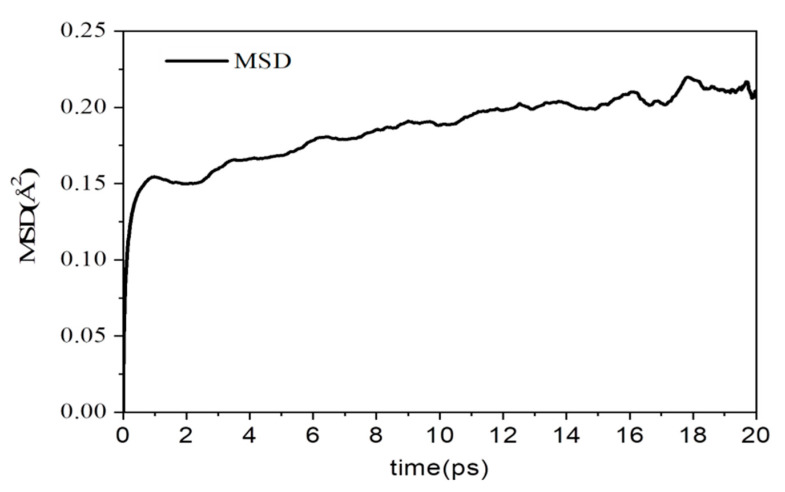
The mean square displacement (MSD) of protons in our simulation system at 423 K over a period of 20 ps.

The mean square displacement (MSD) of the protons during the simulation can be used to provide the dynamic properties of protons. Figure 3 displays an example of the MSD of protons at 423 K (150 °C) over a period of time of 20 ps. The self-diffusion coefficient of proton, 7.05 × 10^−12^ cm^2^ s^−1^, at 423 K was obtained using Equation (1). The proton conductivity calculated by Equation (2) was 1.06 × 10^−3^ Scm^−1^ at 423 K. For comparison, the experimentally measured value was close to 2 × 10^−4^ Scm^−1^ in the PVPA mix of 89 mol% Py composite material [12]. The results show that the MD simulation system we constructed could provide a proton conductivity close to the value of a real composite material. A larger part of the discrepancy between our computational results and the experimental values may be attributed to temperature difference for the proton conductivity increases with temperature.

### 3.2. Pair Correlation Function and Coordination Study

**Figure 4:**
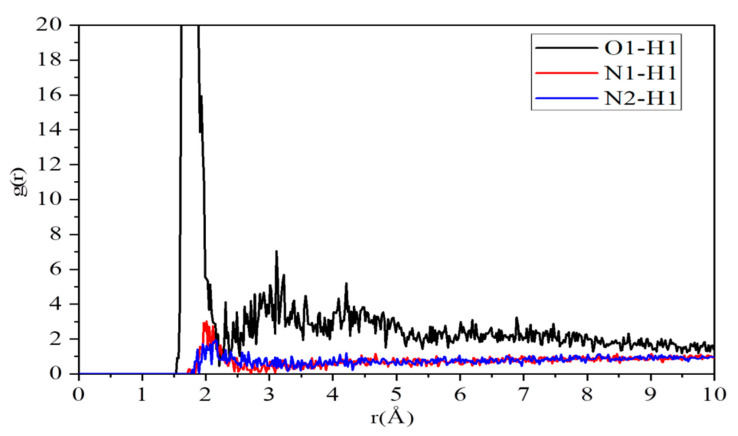
Intermolecular pair correlation functions of O1–H1, N1–H1 and N2–H1.

Intermolecular pair correlation functions of O1–H1 pair, N1–H1 pair, and N2–H1 at 423 K are illustrated in Figure 4. The highest peaks of O1–H1 pair, N1–H1 pair and N2–H1 pair appear at the positions of 1.73 Å, 2.15 Å and 2.01 Å, respectively. All these distances fall within the reasonable range of a hydrogen bond. These results indicate that the protons have strong interaction with the oxygen atoms of PVPA polymers and the nitrogen atoms of pyrazole molecules. As expected, the simulation results confirm that the interaction of O1–H1 pair is stronger than N1–H1 pair and N2–H1 pair.

The fact that there is interaction between protons and the nitrogen atoms of pyrazole molecules indicates pyrazole molecules can attract protons and act as a Bronsted base. The analysis of the coordination numbers provides more details about the distribution of particles in the acid–base composite system. As seen in Table 1, the corresponding coordination numbers of O1–H1 pair, N1–H1 pair, and N2–H1 are 2.74, 0.39, and 0.29, respectively. These results provide quantitative evidence that more protons gather around the oxygen atoms of PVPA polymers than around the nitrogen atoms of pyrazole molecules.

**Figure 5:**
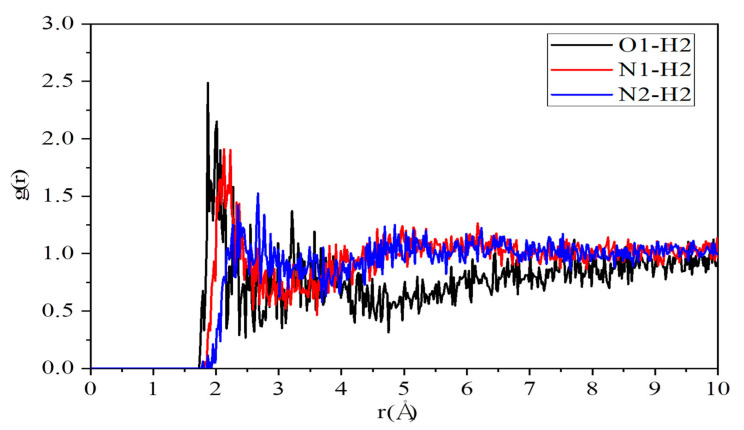
Intermolecular pair correlation functions of O1–H2 pair, N1–H2 pair, and N2–H2.

Figure 5 illustrates intermolecular pair correlation functions of O1–H2 pair, N1–H2 pair, and N2–H2 at 413 K. The highest peaks of O1–H2 pair, N1–H2 pair, and N2–H2 appear at the positions of 2.01 Å, 2.67 Å, and 2.23 Å respectively. All these distances also fall well within the range of a hydrogen bond. These results suggest that the hydrogen bond can form not only between PVPA polymers and pyrazole molecules but also among pyrazole molecules. Moreover, the corresponding coordination numbers of O1–H2 pair, N1–H2 pair, and N2–H2 pair are 0.09, 0.64, and 0.45 as given in Table 1. The values of these coordination numbers show that pyrazole molecules could get too close to each other by relatively strong interaction, hydrogen bond. The network of the hydrogen bond can provide a pathway to transport protons.

### 3.3. Analysis of Proton Trajectories

**Figure 6:**
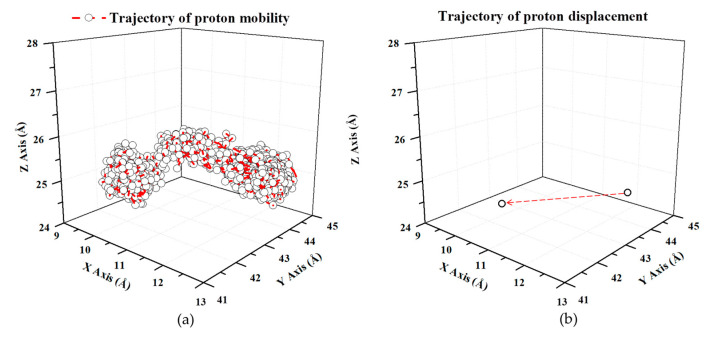
(**a**) The trajectory of a selected proton and (**b**) the displacement of a selected proton, over a simulation run.

The base-doped poly(vinylphosphonic acid) composite can generate free protons. As a result, the PVPA polymer can act as a proton donor, and base such as N-heterocycles can act as a proton acceptor. Therefore, by analyzing the trajectories of protons, it can further provide the process of transport in pyrazole-doped poly(vinylphosphonic acid) system. Figure 6a is an example of the trajectory of a selected proton over a period of 140 ps. The proton moves through a large part of the simulation volume during the simulation time, demonstrating high proton mobility in our simulated system. In addition, the measurement shown in Figure 6b indicates that the selected proton has a displacement of ~3 Å from the first step to the final step during a simulation run. 

Intermolecular pair correlation functions of O1–H1 pair, N1–H1 pair, and N2–H1 pair show the protons have strong interactions with nitrogen atoms of pyrazole molecules and oxygen atoms of PVPA polymer. Therefore, the interactions between particles can drive the proton to mobilize.

**Figure 7:**
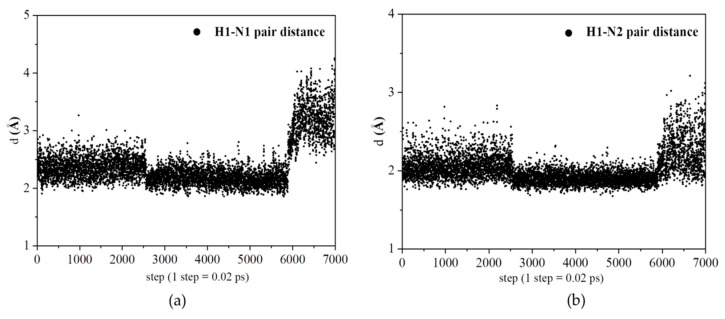
(**a**) The relative distance of a selected proton with N1 (N1 in Py). (**b**) The relative distance of a selected proton with N2 (N2 in Py).

Figure 7a shows the relative distance between a selected proton and a selected N1 atom of pyrazole molecule during a period of 140 ps. The relative distance of the H1–N1 pair changes from about 2 Å to almost 4.3 Å. Figure 7b shows another relative distance between the proton and N2 atom of the same pyrazole molecule during a period of 140 ps. The relative distance of the H1–N2 pair changes from about 2 Å to almost 3.3 Å.

These results indicate the stronger interactions exist between the proton and pyrazole molecule during a period of 120 ps. After the time of 120 ps, the proton is away from the pyrazole molecule.

**Figure 8:**
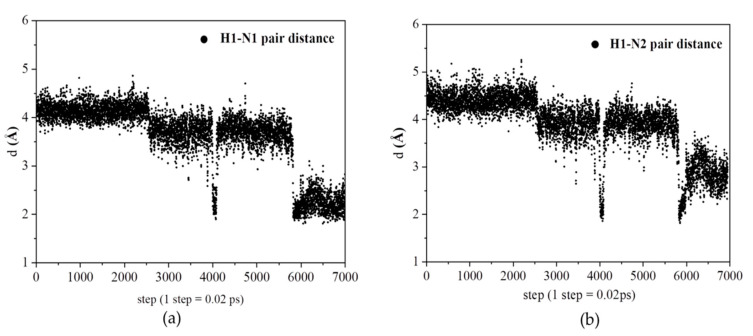
(**a**) The relative distance of a selected proton with N1 (N1 in Py). (**b**) The relative distance of a selected proton with N2 (N2 in Py).

**Figure 9:**
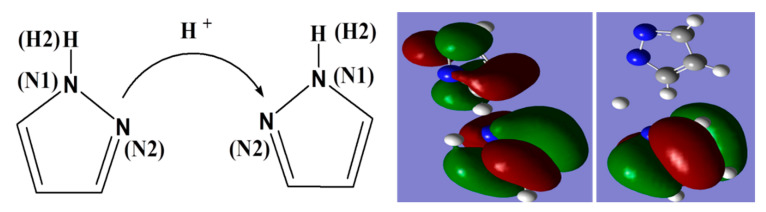
A visual presentation of proton migration along pyrazole groups based on distance analysis. Shown on the right-hand side is the DFT computation result of the protonated and deprotonated pyrazoles, in qualitative agreement with the molecular dynamics simulation result.

Figure 8a,b shows the same proton has strong interactions with nitrogen atoms (N1 and N2) of another pyrazole molecule after the time of 120 ps. The results based on these figures as mentioned above indicate the protons migrate from original pyrazole group to the next one. Based on the distance analysis, the proton migration along Py chain is visualized in Figure 9. This picture is further verified with ab initio computation with two pyrazoles, as shown on the right-hand side of Figure 9, where the protonated and deprotonated pyrazoles are clearly shown. In ab initio computation, as expected, the continuous change of the partial charge on the proton was observed.

**Figure 10:**
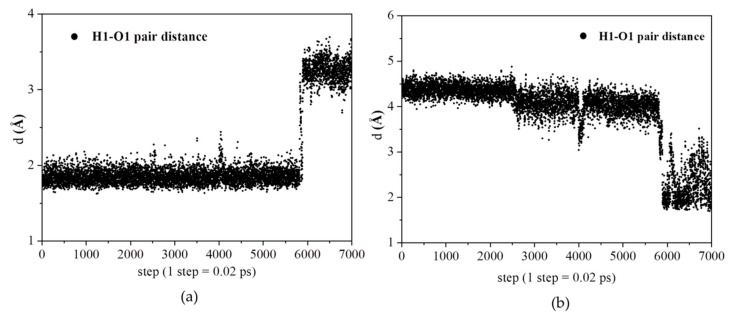
(**a**) The relative distance of a selected proton with ON1 (N1-O1 in PVPA). (**b**) The relative distance of a selected proton with ON2 (ON2 in PVPA).

**Figure 11:**
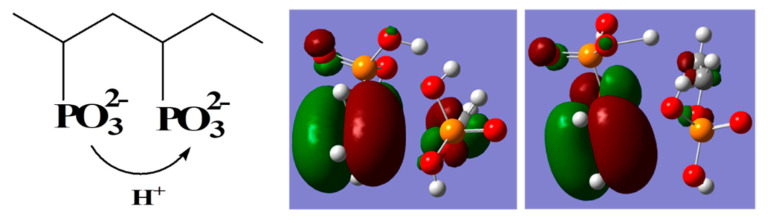
A visual presentation of proton migration along phosphate groups based on distance analysis. Shown on the right-hand side is the DFT computation result of the protonated and deprotonated vinyl phosphates, in qualitative agreement with the molecular dynamics simulation result.

The mobility of proton also can be observed in PVPA polymer chain. Figure 10a shows the relative distance between a selected proton and a selected O1 atom of PVPA during a period of 140 ps. The relative distance of the H1-O1 pair changes from about 1.7 Å to almost 3.7 Å. Figure 10b shows another relative distance between the same proton and another O1 atom of PVPA polymer during a period of 140 ps. The relative distance of the newly formed H1-O1 pair changes from about 4.7 Å to almost 1.5 Å. These results indicate that stronger interactions exist between the proton and O1 atom of PVPA polymer over a period of about 120 ps. After 120 ps, the proton moves away from the O1 atom and hops to the next O1 of PVPA polymer. Figure 10b shows the same proton having strong interactions with another O1 atom of PVPA chain after 120 ps. Hence, the results provide direct evidence that the protons migrate from one site close to an O1 atom of PVPA chain to the next. Therefore, the proton transfer between phosphate groups is shown in Figure 11. Analogous to proton transfer between pyrazoles, this picture is further verified with ab initio computation with two vinyl phosphates, as shown on the right-hand side of Figure 11, where the protonated and deprotonated vinyl phosphates are clearly shown. In ab initio computation, as expected, the continuous change of the partial charge on the proton was observed.

## 4. Conclusions

To elucidate the proton transport mechanism in anhydrous proton exchange membranes, a molecular dynamics simulation based on Dreiding force field has been carried out on a composite system of mixing protons, poly(vinyl phosphonate anion) and pyrazole, as a more realistic representative of anhydrous PEMs used in fuel cells. The intermolecular pair correlation functions and coordination numbers indicate that the appreciable interactions and hydrogen bonding exist among poly(vinyl phosphonate anion) and pyrazole molecules or among pyrazole molecules. The trajectories of the protons confirm that the proton mobility is facilitated by hydrogen bonds forming and breaking. The network of hydrogen bonds can provide a pathway for proton transport. To the best of our knowledge, the proton conductivity of 1.06 × 10^−3^ Scm^−1^ reasonably close to 2 × 10^−4^ Scm^−1^, the experimental data, has never been reached before, demonstrating that the Dreiding force field and the simulation design can quantitatively reproduce the experimental data of real anhydrous PEMs and reveal the proton transport mechanism in them. It is expected that further refinement of simulation design may provide better agreement between simulation and experimental data.

## Figures and Tables

**Table 1:** The maxima of the radial distribution functions and the coordination numbers of the selected atom pairs.

Correlation Pair	Position of Highest Peak	Coordination Number
O1–H1	1.73 Å	2.74
N1–H1	2.15 Å	0.39
N2–H1	2.01 Å	0.29
O1–H2	2.01 Å	0.09
N1–H2	2.67 Å	0.64
N2–H2	2.23 Å	0.45
